# Alpha-pinene alleviates CCl_4_-induced renal and testicular injury in rats by targeting oxidative stress, inflammation, and apoptosis

**DOI:** 10.22038/IJBMS.2024.73116.15890

**Published:** 2024

**Authors:** Fatemeh Noroozi, Masoumeh Asle-Rousta, Rahim Amini, Zeinab Sahraeian

**Affiliations:** 1 Department of Physiology, Zanjan Branch, Islamic Azad University, Zanjan, Iran; 2 Department of Biology, Zanjan Branch, Islamic Azad University, Zanjan, Iran; 3 Nanobiotechnology Research Center, Zanjan Branch, Islamic Azad University, Zanjan, Iran

**Keywords:** Alpha-pinene, Bcl-2-associated X protein, Inflammation, Kidney, Oxidative stress, Testis

## Abstract

**Objective(s):**

Renal and testicular disorders are primarily associated with oxidative damage and inflammation. Here, alpha-pinene (a type of monoterpene) was investigated for its effect on oxidative/nitrosative stress and the expression of inflammatory and apoptotic factors in the kidneys and testes of rats treated with CCl_4_.

**Materials and Methods:**

CCl_4_ was injected intraperitoneally (IP) at a dose of 2 ml/kg (twice a week for six weeks). Alpha-pinene (50 mg/kg/day, IP) was also treated during the same period.

**Results:**

CCl_4_ increased the level of malondialdehyde (*P*<0.01 in the kidney and *P*<0.001 in the testis) and nitric oxide (*P*<0.001 in the kidney and *P*<0.01 in the testis) and decreased the levels of glutathione (*P*<0.05) in the kidneys and testicles of rats. CCl_4_ also reduced the catalase enzyme activity in the kidneys (*P*<0.05) but did not affect its activity in the testis. In addition, CCl_4_ enhanced the mRNA expression of TNF-α (*P*<0.01), nuclear factor-κB (P<0.05), and Bax (*P*<0.05 in the kidney and *P*<0.01 in the testis) and decreased the expression of Bcl-2 (*P*<0.05) in both organs. Alpha-pinene prevented all the mentioned changes, but it did not influence the expression of Bcl-2 in the kidneys of rats receiving CCl_4_.

**Conclusion:**

Alpha-pinene may have the potential to prevent renal and testicular diseases by strengthening the antioxidant system in the kidneys and testis, and inhibiting oxidative/nitrosative stress, inflammation, and apoptosis caused by CCl_4_.

## Introduction

Carbon tetrachloride (CCl_4_) is a toxic organic compound used to cause hepatic fibrosis and cirrhosis in animal models. Studies have shown that CCl_4_ also damages the kidneys, testicles, brain, and lungs (1). As a result, CCl_4_ is used to induce testicular toxicity (2), neurotoxicity (3), hepatotoxicity, and nephrotoxicity (4) as well. Evidence indicates that CCl_4_ toxicity starts with the onset of oxidative stress (2, 5). CCl_4_ is converted into trichloromethyl and trichloromethyl peroxyl radicals in cells, and the production of these compounds leads to lipid peroxidation. Lipid peroxidation in cell membrane structures results in oxidative stress, mitochondrial stress response, and endoplasmic reticulum stress (1). CCl_4_ decreases the activity of superoxide dismutase (SOD), glutathione peroxidase, catalase, and glutathione reductase enzymes, and changes the levels of glutathione (GSH) and malondialdehyde (MDA) in the kidneys and testes (6, 7). On the other hand, this compound induces inflammation by increasing the expression of pro-inflammatory factors such as tumor necrosis factor-α (TNF-α) and interleukin-6 (IL-6) and enhancing the activity of nuclear factor-κB (NF-κB) (7, 8). Apoptosis in liver, kidney, and testis cells is also one of the consequences of exposure to CCl_4_, which is caused by changing the expression of B-cell lymphoma protein 2 (Bcl-2)-associated X (Bax) and Bcl-2 (which, respectively, stimulate and inhibit apoptosis) (6, 9, 10). Considering that the harmful effects of CCl_4_ in different organs of the body are mediated through the induction of oxidative stress, inflammation, and apoptosis, it has been suggested that antioxidants are a suitable option to prevent and reduce the adverse effects caused by this toxin (1).

Terpenes are natural compounds that possess antioxidant potential (11). Alpha-pinene, a monoterpene, is present in conifers and pines, rosemary, eucalyptus, and camphor (12). It has anti-oxidative (13), anti-inflammatory (14), and anti-apoptotic (15) properties. Studies have shown that it provides protection against Alzheimer’s disease (14), epilepsy (16), diabetes (17), myocardial infarction (18), and ischemic stroke (19), by inhibiting oxidative stress, inflammation, and cell death. However, there is no evidence of alpha-pinene’s effects on kidney and testicular damage. Therefore, we conducted a study to investigate its effects on oxidative and nitrosative stress, inflammation, and the expression of apoptotic agents in the kidneys and testicles of male rats receiving CCl_4_.

## Materials and Methods

***Grouping and outline of the experiment***

A total of twenty-four male Wistar rats weighing between 200–220 grams were obtained from Shahid Beheshti University of Medical Sciences (Iran) and housed in the Nanobiotechnology Research Center of Islamic Azad University, Zanjan branch (Iran) under standard laboratory conditions. The procedures for working with animals were approved by the Ethics Committee of IAUZ (code: IR.IAU.Z.REC.1401.036).

The rats were divided into four groups (6 rats in each group). The control group did not receive any treatment. The alpha-pinene (P) group received alpha-pinene (50 mg/kg, intraperitoneally) for six consecutive weeks (19). The CCl_4_ group was also treated with CCl_4_ (30% CCl_4_ in olive oil) at a dose of 2 ml/kg twice a week for six consecutive weeks (20). The CCl_4_. P group received both substances during this period. It is worth mentioning that intraperitoneal administration of CCl_4_ is a more common way to start fibrosis, although oral, subcutaneous, and inhaled treatments are also used (21). As a result, we employed intraperitoneal injection of CCl_4_. Alpha-pinene was purchased from Sigma (USA) and CCl_4_ was acquired from Merck (Germany).

At the end of the sixth week, the animals were sacrificed under anesthesia. The kidneys and testes were removed for biochemical studies (surveying the levels of MDA, nitric oxide, and GSH and measuring the activity of catalase enzyme) and molecular investigations (measurement of the expression of Bax, Bcl-2, TNF-α, and NF-κB by real-time PCR method).

***Biochemical studies***

*Homogenization of liver samples*

For the biochemical studies, we used the right kidneys and the right testes of the animals. We started by homogenizing the samples in Tris buffer (0.025 M, pH 7.5), followed by centrifuging them at 12000 g for 20 min at 4 °C. The resulting supernatant was then used for antioxidant tests. The protein concentration was determined using Lowry’s method (22). Then, we used homogenized testis and kidney tissues to measure the levels of MDA, NO, and GSH, as well as the activity of the catalase enzyme, by using kits provided by Arsam Farazist Company (Urmia, Iran). The measurements were carried out according to the instructions provided with the kits.

*Measurement of MDA levels*

To assess the level of lipid peroxidation, the MDA content was measured, which is based on the reaction of MDA with thiobarbituric acid (TBA) and the formation of a pink complex. Briefly, 250 μl of homogenized tissue was mixed with 500 μl of trichloroacetic acid solution and placed in a hot water bath at a temperature of 95 °C for 15 min. Then it was centrifuged (14,000 g) for 5 min. Two hundred fifty microliters of TBA solution were added to the supernatant, and after being placed in a hot water bath at 95 °C (for 10 min), its absorbance was read at a wavelength of 532 nm. The concentration of MDA was reported based on nmol/mg of protein (23).

*Estimation of NO levels*

To measure NO, the amount of nitrite (NO2^-^), which is one of the two products of NO, was measured with the Arsam Farazist kit. This test is based on the reaction of nitrite with sulfanilamide and N-(1-naphthyl)ethylenediamine dihydrochloride (NED) under acidic conditions and the production of an azo compound, which can be seen in pink color (Gries reaction). To determine the amount of nitrite, first, 20 μl of homogenous tissue was mixed with 880 μl of distilled water. In the next step, 50 μl of sulfanilamide was added and incubated for 5 min at room temperature in the dark, and at the end, after adding 50 μl of NED reagent, its absorbance was read at a wavelength of 520 nm. The content of NO was expressed as nmol/mg protein (24).

*Assessment of GSH content*

The concentration of GSH was determined by mixing 100 μl of the tissue homogenate with 300 μl of the diluted buffer. Next, 100 μl of sulfosalicylic acid was added to it and incubated for 12 min on ice. The solid was removed by a centrifugation process of 12,000 g for 5 min. Then, 400 μl of reaction buffer and 100 μl of 5,5ʹ-dithiobis-(2-nitrobenzoic) acid were added to the supernatant. Finally, the absorbance was read at 412 nm wavelength, and the GSH amount was expressed as μg/mg protein (25).

*Catalase activity assay*

The activity of catalase is determined based on the reaction of this enzyme with methanol and the production of formaldehyde. The chromogenic reagent formed a heterocyclic ring with formaldehyde, which changed from colorless to purple. First, 200 μl of reaction buffer was mixed with 150 μl of methanol and 30 μl of H2O2 and gently shaken. Then, 50 μl of homogenate was added, and it was incubated for 20 min in the dark. Next, 150 μl of potassium hydroxide solution and 150 μl of chromogenic reagent were added, and the mixture was incubated for 10 min. After this period, 150 μl of potassium periodate was added to the samples, and after mixing, they were centrifuged (10,000 g for 10 min). Finally, the absorbance of the samples was read at 550 nm, and the amount of catalase enzyme activity was expressed as U/mg protein.

***Real-time PCR***

The mRNA expression of TNF-α, NF-κB, Bax, and Bcl-2 in the left testis and left kidney of five rats from each group was determined by real-time PCR. 

Total RNA was extracted using the GeneAll RNA extraction kit (Seoul, Korea). The RNA concentration and quality were determined using a spectrophotometer at 260 nm by the optical density of A260/A280. Next, 1 μg of RNA from each sample was used to synthesize cDNA using the ExcelRT™ Reverse Transcription kit (SMOBIO, Taiwan). Real-time polymerase chain reaction was performed with an ABI StepOnePlus thermocycler (Applied Biosystems, USA) using RealQ Plus 2x Master Mix Green High RoxTM (Ampliqon, Denmark). Melting curve analysis was performed based on a denaturation step at 95 °C for 15 sec followed by 40 cycles at 95 °C for 20 sec and 60 °C for 60 sec. To ensure accuracy, the experiment was repeated twice for each sample. GAPDH was used as the internal reference gene, and the relative expression levels of target genes relative to the control group were calculated using the 2^-ΔΔCT^ comparative expression method (26). Primers were purchased from Bioneer (Korea) according to Table 1.

***Statistical analyses***

Data were analyzed statistically using a one-way ANOVA test in the SPSS software version 16. The results were expressed as mean ± standard error of the mean (SEM). The differences among groups were detected by the Tukey test. *P*<0.05 was considered significant.

## Results

***The effect of alpha-pinene on oxidative and nitrosative stress in the kidneys and testes of rats receiving CCl***_4_

**Figure 1:**
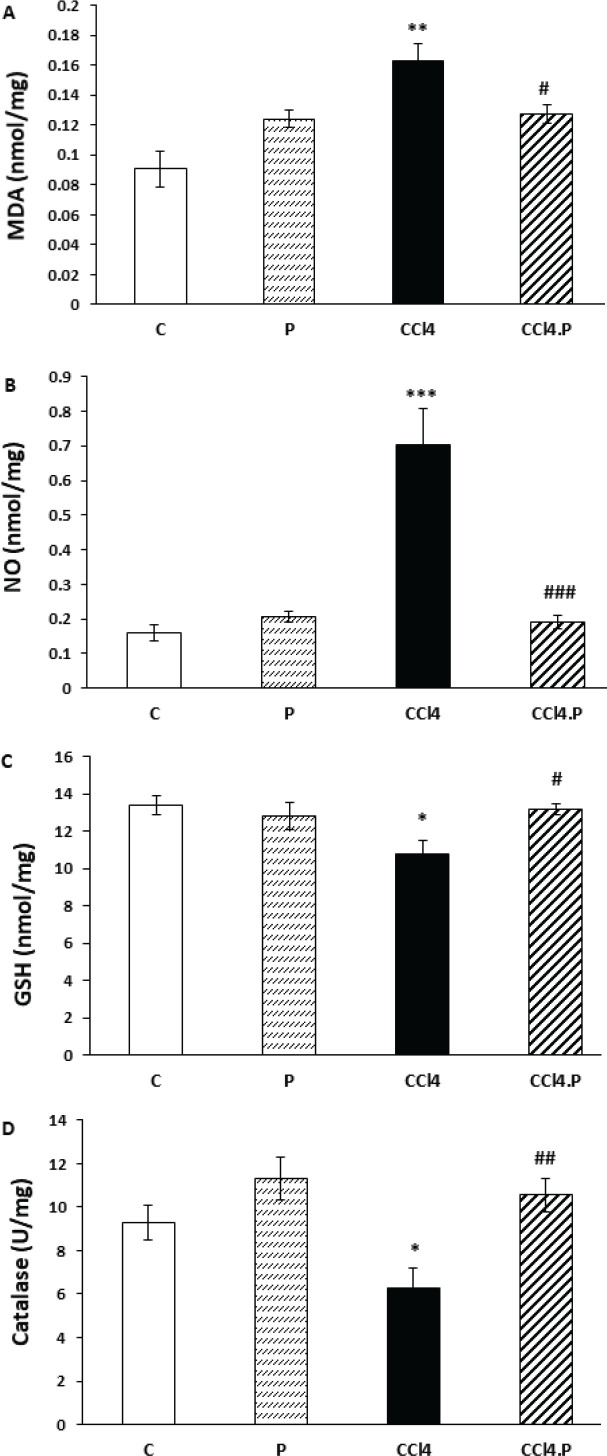
Effect of alpha-pinene on the level of (A) MDA, (B) NO, (C) GSH, and (D) catalase enzyme activity in the kidneys of rats receiving CCl4

**Figure 2:**
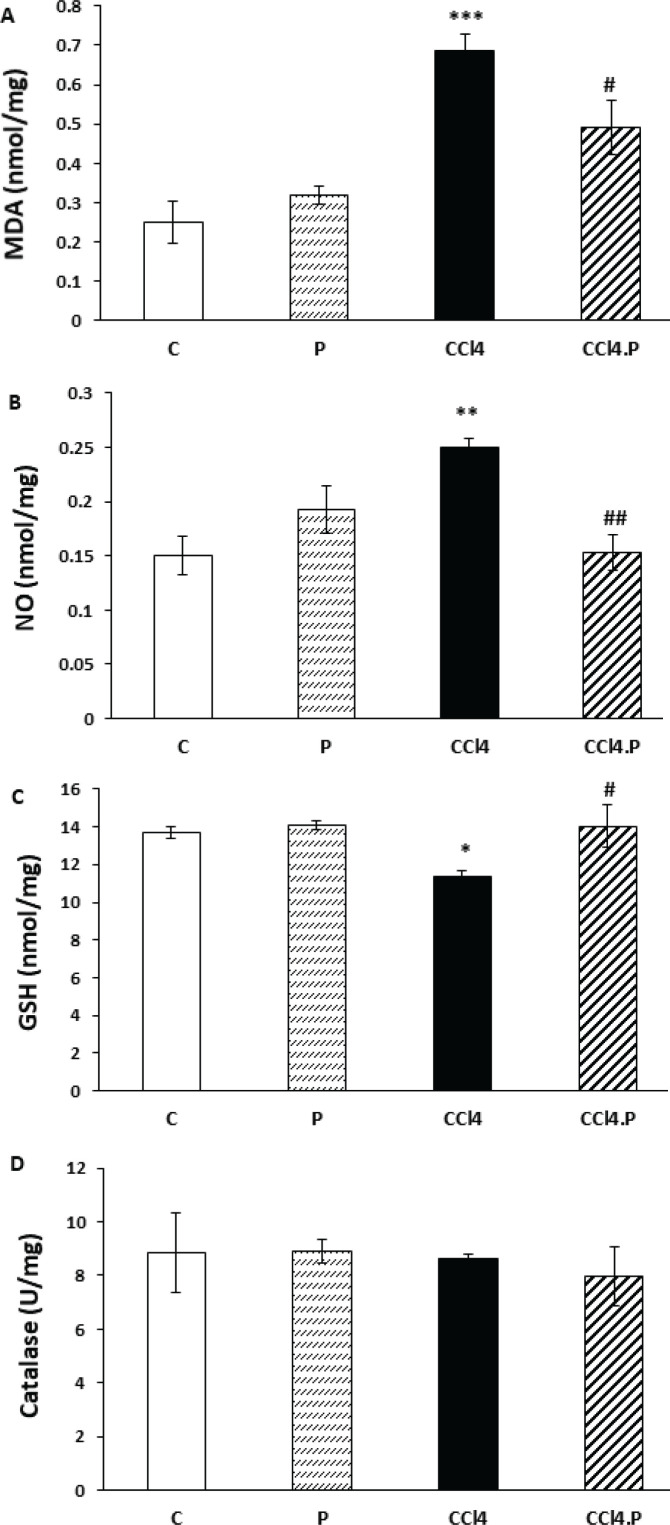
Effect of alpha-pinene on the level of (A) MDA, (B) NO, (C) GSH, and (D) catalase enzyme activity in the testis of rats receiving CCl4

The study found that injecting CCl_4_ into the peritoneum significantly increased the levels of malondialdehyde (MDA) in the kidneys and testes of rats compared to the control group (*P*=0.001 and *P*=0.000, respectively). However, the levels of MDA in the kidneys and testes of rats in the CCl_4_.P group were significantly lower than in the CCl_4_ group (*P*=0.044 and *P*=0.021, respectively), as shown in Figures 1A and 2A. 

Furthermore, the levels of nitric oxide (NO) in the kidneys and testicles of the CCl_4_ group were significantly higher compared to the control group (*P*=0.000 and *P*=0.001, respectively). Treatment with alpha-pinene successfully prevented the increase of NO in the CCl_4_.P group, as the level of NO in the kidneys and testes of CCl_4_.P animals was significantly lower than in the CCl_4_ group (*P*=0.000 and *P*=0.001, respectively), as shown in Figures 1B and 2B. 

The study also found that the GSH content in the kidneys and testes of rats in the CCl_4_ group was significantly lower (*P*=0.028 and *P*=0.034, respectively) compared to the control group. However, treatment with alpha-pinene caused the level of GSH in the kidney (*P*=0.025) and testes (*P*=0.018) of CCl_4_.P animals to be significantly higher than in the CCl_4_ group, as shown in Figures 1C and 2C. 

Moreover, the study found that CCl_4_ led to a significant decline in catalase activity in rat kidneys (*P*=0.033). However, alpha-pinene prevented the decrease in the activity of this enzyme in the kidney of CCl_4_.P animals, so this factor in the CCl_4_.P group was significantly higher than in the CCl_4_ group (*P*=0.008) (Figure 1D). The catalase activity in the testes of all studied groups showed no significant difference, as shown in Figure 2D. 

Finally, none of the factors studied in biochemical tests were significantly different in group P from the controls, as shown in Figures 1 and 2.

***Effect of alpha-pinene on TNF-α/NF-κB signaling in the kidneys and testes of rats receiving CCl***_4_

**Figure 3:**
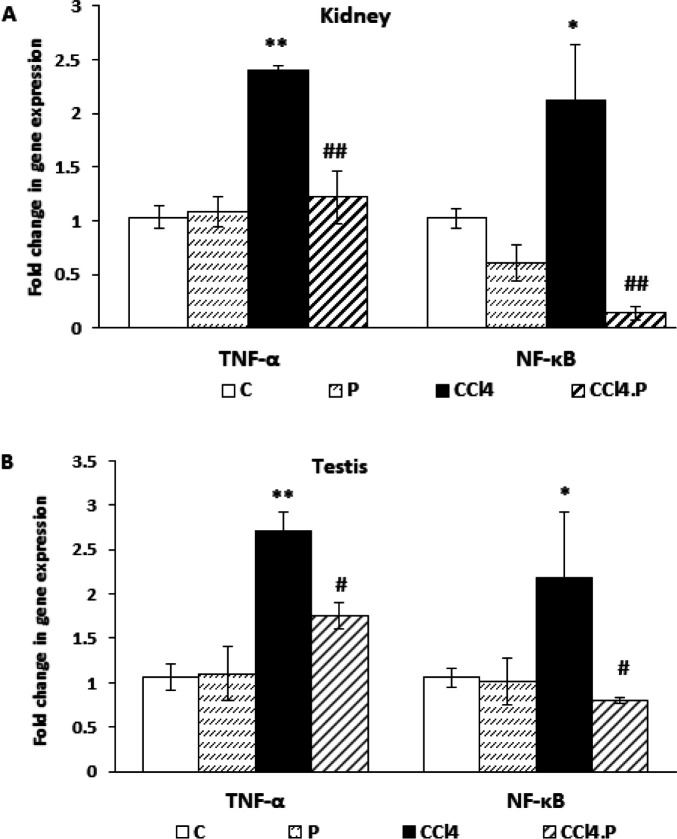
Effect of alpha-pinene on the mRNA expression of TNF-α and NF-κB in the (A) kidney and (B) testis of rats receiving CCl4

The results of the real-time PCR test showed that injection of CCl_4_ increased the expression of TNF-α in the kidneys (*P*=0.001) and testes (*P*=0.002). CCl_4_.P animals had lower mRNA expression of this inflammatory mediator in the kidneys and testes than CCl_4_ rats (*P*=0.002 and *P*=0.022, respectively) (Figure 3A, B).

Similarly, CCl_4_ increased NF-κB mRNA expression in the kidneys (*P*=0.033) and testes (*P*<0.036) compared to the control group. Alpha-pinene inhibited the increase in the expression of this transcription factor in the kidneys and testes of rats receiving CCl_4 _(*P*=0.002 and *P*=0.015, respectively) (Figure 3A, B).

The expression of TNF-α and NF-κB was not significantly different in either organ in the P group compared to the controls (Figure 3).

***Effect of alpha-pinene on the expression of Bax and Bcl-2 in the kidneys and testes of rats receiving CCl***_4_

**Figure 4:**
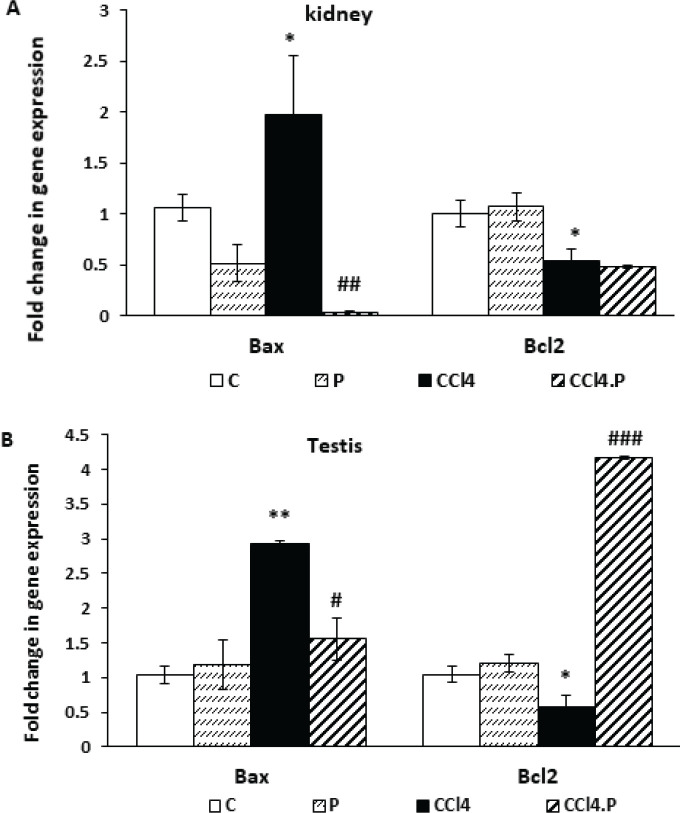
Effect of alpha-pinene on the mRNA expression of Bax and Bcl-2 in the (A) kidneys and (B) testes of rats receiving CCl4

In rats that were treated with CCl_4_, the expression of Bax was significantly higher in the kidneys and testes compared to the control group (*P*=0.037 and *P*=0.002, respectively). However, when these rats were treated with alpha-pinene, the expression of Bax was decreased in the CCl_4_.P group compared to the CCl_4_ group (*P*=0.001 and *P*=0.013, respectively) (Figure 4).

Additionally, CCl_4_ injection caused a significant decrease in the expression of Bcl-2 in the kidneys and testes of rats (*P*=0.035 and *P*=0.047, respectively) compared to the control group. Alpha-pinene treatment did not prevent the reduction of Bcl-2 expression in the kidneys of rats injected with CCl_4_ (*P*=0.605). However, it did cause the expression of this anti-apoptotic factor to be significantly higher in the testes of the CCl_4_.P group compared to the CCl_4_ group (*P*=0.000) (Figure 4).

It is worth noting that the expression of Bax and Bcl-2 in the kidneys and testes of P-group rats was not significantly different when compared to the control group (Figure 4).

## Discussion

CCl_4_ is a potent toxin that induces oxidative stress, inflammation, and apoptosis in the liver, kidney, testis, lung, and brain (1, 3, 27, 28). Our study showed that intraperitoneal injection of CCl_4_ after six weeks increased the levels of MDA and NO, decreased the levels of GSH in the kidneys and testicles, and reduced the activity of catalase in the kidney of rats. CCl_4_ also increased the expression of TNF-α, NF-κB, and Bax in these organs and decreased the expression of Bcl-2.

Oxidative stress plays an essential role in kidney and testicular dysfunction. It leads to a decrease in sperm count and fertility, damage to sperm DNA, and lipid peroxidation in the sperm membrane. The kidneys are also subjected to oxidative stress, which results in modifications in the structure of the nephrons, which in turn plays an essential role in the progression of both acute and chronic kidney ailments. Therefore, strengthening the antioxidant system has been introduced as a solution to curb nephrotoxicity and testicular toxicity caused by CCl_4_ (1). In this research, to investigate the effect of alpha-pinene on oxidative stress caused by CCl_4_ injection in the kidneys and testes, we injected this monoterpene at a dose of 50 mg/kg intraperitoneally to the animals. The dose of alpha-pinene was determined based on the report by Khoshnazar *et al*. (19) and Khan-Mohammadi-Khorrami *et al*. (14), which demonstrated the ability of alpha-pinene to reduce oxidative stress in the hippocampus, cortex, and striatum of ischemia and Alzheimer’s disease model rats. The six-week course of alpha-pinene administration in rats receiving CCl_4 _prevented the increase in MDA and NO concentrations and the decrease of GSH concentrations in both tissues. It also prevented the decrease in catalase activity in the kidneys. These results indicate that alpha-pinene prevented oxidative stress in the kidneys and testes of CCl_4_-injected animals.

No changes were observed in the catalase activity of the testicles in the CCl_4_ group or other groups examined in the present study. Previous studies have reported a decrease in the activity of this enzyme in the testis after intraperitoneal injection of a higher dose (3 ml 50% CCl_4_) than that used in this study (29) and after a longer duration of treatment (12 weeks) (30). Therefore, the use of a different dose and duration of CCl_4_ treatment in this study may have reduced the catalase activity in the testis.

There is a close relationship between oxidative stress and kidney inflammation. Inflammatory factors, which are produced to repair tissue damage caused by oxidative stress, are themselves considered a source of free radical production and eventually, disrupt the function of nephrons. For example, inflammatory factors reduce the glomerular filtration rate. Oxidative stress and inflammation in the kidney act cyclically and reinforce each other (31). In the testis, reactive oxygen species (ROS) contribute to the excessive production of free radicals and induce inflammatory responses. In the testis, macrophages and Sertoli and Leydig cells make cytokines like TNF-α, IL-1, and IL-6. In physiological conditions, the amount of ROS is low because the excess ROS is removed by the endogenous antioxidant system, which maintains the seminal redox balance. However, inflammatory damage in the testis leads to increased production of ROS (32). Since alpha-pinene, by strengthening the antioxidant system, prevented the incidence of oxidative stress in the kidneys and testicles of CCl_4_.P animals, we predicted that it would also reduce the expression of TNF-α, and the results of real-time PCR confirmed this prediction. NF-κB, which plays a substantial role in the development and progression of inflammatory processes, is activated by various stimuli, including TNF-α, and increases the expression of inflammatory mediators, including TNF-α, IL-1, IL-6, and IL-8 (33). Therefore, the decrease in NF-κB expression likely was the outcome of a reduction in TNF-α mRNA expression in the kidneys and testes of CCl_4_.P group rats. As a result, alpha-pinene showed its anti-inflammatory effect by inhibiting TNF-α/NF-κB signaling. It is better to examine TNF-α and NF-κB protein expression. In confirmation of these results, the anti-inflammatory effect of alpha-pinene has been reported in several *in vitro *and* in vivo* studies (14, 17, 19, 34). 

Activation of NF-κB leads to the activation of inducible nitric oxide, which increases the production of NO (35). The inhibition of NOS prevents hepatotoxicity caused by CCl_4_ (36). Therefore, alpha-pinene’s ability to reduce NO levels may be due to its inhibitory effect on the expression of NF-κB in the kidneys and testes of rats receiving CCl_4_. Measuring the activities of NF-κB and inducible NOS can also be useful.

Oxidative stress is associated with the development of apoptosis. ROS leads to apoptosis by increasing the activity of NF-κB, which has been introduced as a biomarker of oxidative stress (37, 38). Studies have shown that inhibition of NF-κB prevents the apoptosis of germ cells in seminiferous tubules (39). In the kidney, NF-κB is activated following the increase of TNF-α and leads to the development of inflammation and fibrosis (40).

Research has shown that CCl_4_ activates the internal pathway of apoptosis in hepatocytes by increasing the expression of the pro-apoptotic protein Bax and decreasing the expression of the anti-apoptotic protein Bcl-2 (one of the proteins found in the mitochondrial membrane) (41) which is consistent with the results of our research. We observed that alpha-pinene decreased the expression of Bax in both organs and significantly increased the expression of Bcl-2 in the testis of CCl_4_.P animals. In confirming the results of this section, we can refer to the report of Karthikeyan *et al*. (15). They demonstrated that alpha-pinene prevents apoptosis in human skin epidermal keratinocytes exposed to ultraviolet-A by regulating the expression of Bax/Bcl-2. Although alpha-pinene did not alter the expression of Bcl-2 in the kidney of rats injected with CCl_4_, it significantly reduced the level of Bax, leading to a decrease in the ratio of Bax/Bcl-2 in the kidney of animals in the CCl_4_.P group. This decrease in the ratio of Bax/Bcl-2 plays an important role in enhancing the kidney cells’ resistance to apoptotic conditions, as reported in previous studies (42). Examining Bax and Bcl-2 protein levels in different groups can clarify the effectiveness of alpha-pinene in inhibiting apoptosis.

The protective effect of some terpenes such as limonene (43, 44), eugenol (45, 46), and linalool (47, 48) has been confirmed in the kidney and testicle, but so far there has been no report on the protective effect of alpha-pinene in these organs. However, researchers (49) showed that *Juniperus communis* extract prevented lipopolysaccharide-induced kidney damage in rats. Alpha-pinene is the main component of this plant extract. Recently, the protective effect of *Rosmarinus officinalis* L. extract (which contains abundant amounts of alpha-pinene) against etoposide-induced testis failure was also reported (50). 

**Table 1:** The primer sequences of the related genes used in the real-time PCR

**Gene**	**Forward primers sequence (5'–3')**	**Reverse primers sequence (5'–3')**
**TNF-α**	CACGGGAGCCGTGACTGTA	TCCAAGCGAACTTTATTTCTCTCA
**NF-κB**	CATGGCAGACGACGATCCTT	TGGAGTGAGTCAAAGCAGTATTCAA
**Bax**	CGTGGTTGCCCTCTTCTACT	TCACGGAGGAAGTCCAGTGT
**Bcl-2**	GGGATGCCTTTGTGGAACTA	CTCACTTGTGGCCCAGGTAT
**GAPDH**	GCTACACTGAGGACCAGGTTGTCT	CCCAGCATCAAAGGTGGAA

Data are presented as mean±SEM; n=6. * *P*<0.05, ** *P*<0.01, and *** *P*<0.001 compared with Control group and # *P*<0.05, ## *P*<0.01, and ### *P*<0.001 compared with CCl4 group

C: Control, P: alpha-Pinene, CCl4: Carbon tetrachloride, CCl4.P: Carbon tetrachloride-alpha-Pinene; MDA: Malondialdehyde; GSH: Glutathione

Data are presented as mean±SEM; n=6. * *P*<0.05, ** *P*<0.01, and *** *P*<0.001 compared with the Control group and # *P*<0.05 and ## *P*<0.01 compared with CCl4 group

C: Control, P: alpha-Pinene, CCl4: Carbon tetrachloride, CCl4.P: Carbon tetrachloride-alpha-Pinene; MDA: Malondialdehyde; GSH: Glutathione

Data are presented as mean±SEM; n=5. * *P*<0.05 and ** *P*<0.01 compared with the Control group and # *P*<0.05 and ## *P*<0.01 compared with CCl4 group

C: Control, P: alpha-Pinene, CCl4: Carbon tetrachloride, CCl4.P: Carbon tetrachloride-alpha-Pinene.

Data are presented as mean±SEM; n=5. * *P*<0.05 and ** *P*<0.01 compared with the Control group and # *P*<0.05, ## *P*<0.01, and ### *P*<0.001 compared with CCl4 group. C: Control, P: alpha-Pinene, CCl4: Carbon tetrachloride, CCl_4_.P: Carbon tetrachloride-alpha-Pinene

## Conclusion

The results of the present study indicate that alpha-pinene ameliorates oxidative stress, inflammation, and apoptosis caused by CCl_4_ in the testis and kidney. It appears this monoterpene is a suitable option for preventing and treating kidney and testicular diseases. Our findings need to be supported by additional research, including histological examinations.

## Authors’ Contributions

F N performed the experiments, collected the data, and wrote the manuscript. M AR conceived and designed the experiments, analyzed the data, and revised the manuscript. R A and Z S conceived and designed the experiments. 

## Funding Statement

The authors received no financial support for this research work.

## Conflicts of Interest

None.
